# Percutaneous Bone Marrow Transplantation Using Fractional Ablative Erbium:YAG Laser

**DOI:** 10.1371/journal.pone.0093004

**Published:** 2014-03-25

**Authors:** Luis Rodriguez-Menocal, Marcela Salgado, Stephen Davis, Jill Waibel, Arsalan Shabbir, Audrey Cox, Evangelos V. Badiavas

**Affiliations:** 1 Department of Dermatology and Cutaneous Surgery, University of Miami Miller School of Medicine, Miami, Florida, United States of America; 2 Interdiscipinary Stem Cell Institute, University of Miami Miller School of Medicine, Miami, Florida, United States of America; Wake Forest Institute for Regenerative Medicine, United States of America

## Abstract

Topical application of therapeutic agents has been a mainstay in Dermatology for the treatment of skin disorders but is not commonly used for systemic delivery. For a topically applied agent to reach distant body sites it must first overcome the barrier function of the skin and then penetrate into deeper structures before reaching the systemic circulation. This has limited the use of topically applied agents to those having specific charge, solubility and size restrictions. Pretreatment of the skin with ablative fractional laser appears to enhance the uptake of some topically applied drugs but the ability to effectively deliver agents to distant sites is largely unproven. In this report we used a fractional ablative Erb:YAG (Erbium/Yttrium Aluminum Garnet) laser to facilitate the transfer of bone marrow stem cells through the skin in a murine bone marrow transplant model. Chimerism could be detected in the peripheral blood of recipient C57BL/6 mice that were pretreated with ablative fractional laser and had topically applied enhanced green fluorescent protein (GFP) labeled bone marrow cells from syngeneic donor transgenic mice. This study indicates that fractional laser can be used to deliver stem cells through the skin and remain functionally intact.

## Introduction

Stem cell based therapies have the potential to address many disorders that currently have few if any therapeutic alternatives [1]–[3]. There are nevertheless special considerations that must be taken into account when considering cell-based therapeutics. Cells have several distinct properties that differ significantly from traditional agents such as their relatively large size and their rather fragile nature. The most common means of delivering cells systemically has been by intravenous infusion for purposes such as blood cell transfusion. For stem cell based therapies directed at specific tissue repair, a more localized delivery of cells might be preferred but how these locally administered stem cells will behave and where they will engraft is in many cases uncertain. Injection and surgical implantation techniques have been used when trying to administer cells locally to specific sites. With injections, the pressure gradient created during injection can cause significant damage to cells. The needle diameter chosen for injection also has to be carefully considered so cells are not damaged shearing forces. Alternatively, cells may also be delivered surgically by placement into the surgical field either alone or by incorporating them into a matrix material. Surgical techniques will however require compatibility with the wound healing process and embedding cells into matrices may impact their mobilization to targeted tissues. Developing unique methods of cell delivery will broaden the way in which cells can be used therapeutically. Delivering cells directly to broad areas at predetermined depths is also not possible with these techniques. We have examined the use of factional lasers to create micro-channels in the skin that may be used as conduits for cell delivery locally to tissues and also allow for systemic administration [4]–[6]. Fractional lasers can create microchannels and depending on the laser's parameters the size and depth of these channels can be controlled [7], [8]. Nano and microparticles have been shown to be efficiently administered to depths down to 230 μm by using fractional lasers and these particles have been shown to stay in the dermis for longer than 1 month [9]. In this report we describe the delivery of lineage negative (Lin^−^) bone marrow cells to the skin of irradiated mice using an ablative fractional laser with the goal of achieving functional bone marrow transplantation. Using fractional lasers, we have demonstrated that stem cells can be delivered to the skin, become systemically distributed, engraft into distant organs and remain functionally intact. The cells were given at predetermined depths and could cover broader areas in a more uniform fashion.

While this study may not suggest this model as an alternative for bone marrow transplantation, it does provides proof of concept that cell delivery by fractional lasers is a viable option for the delivery of intact functional cells, even to distant sites.

## Materials and Methods

### Animals

All animals and procedure were approved by the Institutional Animal Care and Use Committee (IACUC) at University of Miami/Miller School of Medicine. Four week- old female recipient C57BL/6J or NOD/SCID mice were obtained from Jackson lab (Bar Harbor, ME, USA). Syngeneic male C57BL/6-Tg (UBC-GFP)30Scha/J mice were chosen as donors for bone marrow cells.

### Irradiation

Recipient mice were placed in a ventilated acrylic container (28 cm diameter, 8 cm depth) between opposing gamma ray irradiation plates in a GAMMACELL50 irradiator. Total body irradiation (TBI) was perfomed before laser exposure and transplantation.

Mice were irradiated to at doses ranging from 200 Centigray (cGy) to 1,000 cGy to fully or partially ablate the recipient's bone marrow and create space for engraftment of donor cells. NOD/SSCID mice received a single dose of 400 cGy. C57BL/6 mice were treated with higher doses (400–1000 cGy) typically fractionated into two doses given 24 hours apart.

### Fractional Laser Treatment

Following irradiation, mice were shaved using a motorized electric clipper and carefully inspected to be sure that only hair was shaved without injury to the underlying skin. Once mice were shaved, they were treated with a Sciton Er/YAG fractional laser. Laser parameters varied from 35 to 500 mj at 11–22% density with no coagulation. Depths of laser channels were determined to be between 35–500 microns depending on the settings. An articulated arm was used to deliver the laser beam onto the skin surface. The handpiece was able to create microscopic treatment zones. The dimension of this treatment area was 1 cm^2^.

### Isolation of Fresh Bone Marrow and Lineage Negative Cells (Lin^−^)

Isolation of fresh bone marrow cells by grinding tibiae, femurs, spines and iliac crest from male C57BL/6-Tg (UBC-GFP) mice using a sterile mortar and pestle as previously described [10].

Lin^−^ cells were separated from whole bone marrow using a mouse cell enrichment kit (Myltenyc Biotechnology, USA). For depletion, cells are magnetically labeled with a cocktail of biotinylated antibodies against a panel of lineage antigens (CD5, CD45R [B220], CD11b, Anti-Gr-1 [Ly- 6G/C], 7–4, and Ter-119 antibodies) and Anti-Biotin MicroBeads that identify differentiated cells. After repeated wash to remove excess antibodies, the cells were incubated with magnetized microbeads that bind and eliminate the antigen-bound antibodies. Unbound lin^−^ cells were purified by magnetic separation using AutoMacs (Myltenyi Biotec Inc, Auburn CA) as a negative fraction under is depleted program. The lin^−^ cells were counted in a hematocytometer

Prior to delivery, the bone marrow cells were washed twice and suspended in PBS containing 2% heat inactivated FBS and 5 mM EDTA

### Bone Marrow Cell Delivery

**Figure 1:**
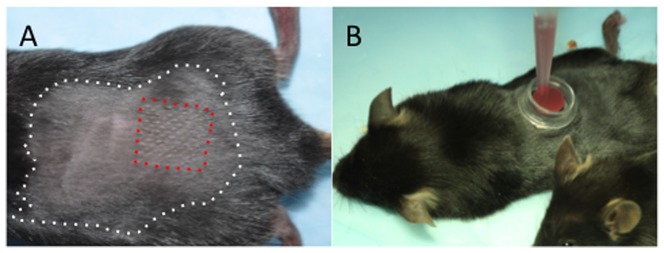
Application of cells following fractional ablative Erb:YAG laser. A) Irradiated C57/BL6 mouse. The white (outer) dashed line highlights the area that was shaved and the red (inner) dashed line highlights the laser treated area. B) Adhered chamber being filled with stem cell suspension.

One day after the last irradiation the laser treatment was performed on the backs of recipient mice. Immediately after fractional laser treatment, a cylindrical chamber that was cut from a 1.5 ml polypropylene micro centrifuge tube was adhered to the laser treatment site (Figure 1) with Dermabond Topical Skin Adhesive (Ethicon, San Lorenzo, Puerto Rico). Approximately 150 μl of fluid containing 0.75×10^6^ lineage negative GFP^+^ bone marrow cells were placed within per chamber and the surface of the chamber sealed with tegaderm. Once sealed, the chambers were no longer manipulated. The chambers were noted to stay in place for at least 24 hours, after which they lost some adherence to the skin.

Control mice were; *i*) not irradiated and administered cells by laser, *ii*) irradiated and given an equivalent dose of Lin^−^ bone marrow cells by tail vein injection, *iii*) irradiated and not given bone marrow cells or *iv*) not irradiated and not given bone marrow cells.

### Flow Cytometry Analysis

Three to four weeks after delivery of donor cells, blood was collected from the tail vein of treated mice to determine their level of chimerism in circulating blood cells. Heparinized mouse blood (0.3 mL) samples were centrifuged at 1,400 g for 5 min at 4°C, and each blood sample was incubated with 3 mL of NH4Cl, pH 7.2, for 10 min at room temperature to lyse the red blood cells. The remaining leukocytes were washed with phosphate-buffered saline (PBS), pH 7.4. As donor cells and their progeny are natively fluorescent (constitutively expressing GFP) labeling of cells with antibody prior to analysis was not required. Samples were analyzed on a BD LSR II Flow Cytometer (Becton, Dickenson and Company, Franklin Lakes, NJ).

### DNA Isolation

DNA was isolated from blood samples using a QIAamp DNA Blood Mini Kit (QIAGEN, Valencia, CA) as per the manufacturers instructions. Liver, spleen and and skin tissues samples were snap frozen in liquid nitrogen and stored at −70°C. Samples were reduced to a fine powder using a stainless steel pulverizer (Biopulverizer; BioSpec Products,Inc., Bartlesville, OK) that had been prechilled in liquid nitrogen.

Pulverized material was immediately transferred to lysis buffer (RNeasy Protect; Qiagen, Valencia, CA) containing mercaptoethanol and further homogenized using a rotor (Pro 200; Pro Scientifc, Oxford, CT) at maximum speed for 1 minute, and stored at −70°C.

### Nested-PCR Analysis

DNA was extracted from blood cells and tissue of recipient mice was prepared as described above and analyzed for the presence of donor specific sequences by Polymerase Chain Reaction (PCR). The reaction was carried out in a Bio-Rad C1000 thermal cycler (Bio-Rad, Hercules, CA) using the Platinum Blue PCR supermix protocol (Invitrogen, Carlsbad, CA) according to the manufacture's protocol. The GFP sequence was detected by a nested-PCR procedure using sequence specific primers (GFP primers: fwd: 5′-AAG TTC ATC TGC ACC ACC G- 3′; rev, 5′-TCC TTG AAG AAG ATG GTG CG-3′. Presence of the Y chromosome was demonstrated using a polymerase chain reaction (PCR) assay with Sry3-sequence (forward primer CGTGGTGAGAGGCACAAGTT; reverse primer ATGGCATGTGGGTTCCTGTC according to primer-blast software NCBI. Similar concentrations of DNA were loaded on each sample tested.

As a control, GAPDH primers were used (FWD, 5′- AACTTTGGCATTGTGGAAGG-3′; REV, 5′-ACACATTGGGGGTAGGAACA-3′) with the annealing temperature at 57 °C for 28 cycles. The PCR products were separated in a 2.0% agarose gel and visualized by ethidium bromide staining.

## Results

### Application of Bone Marrow Cells

After irradiation, mice were treated with fractional Erb:YAG laser to create laser conduits (Figure 1) and chambers were applied to the backs of mice. Once loaded with cells, they chambers were sealed using an occlusive dressing (Tegaderm). All chambers remained intact overnight with most remaining adhered for more than 24 hours. No evidence of wounding related to applying the chambers was noted in any animal.

### Generation of EGFP Chimeric NOD/SCID Mice

**Figure 2:**
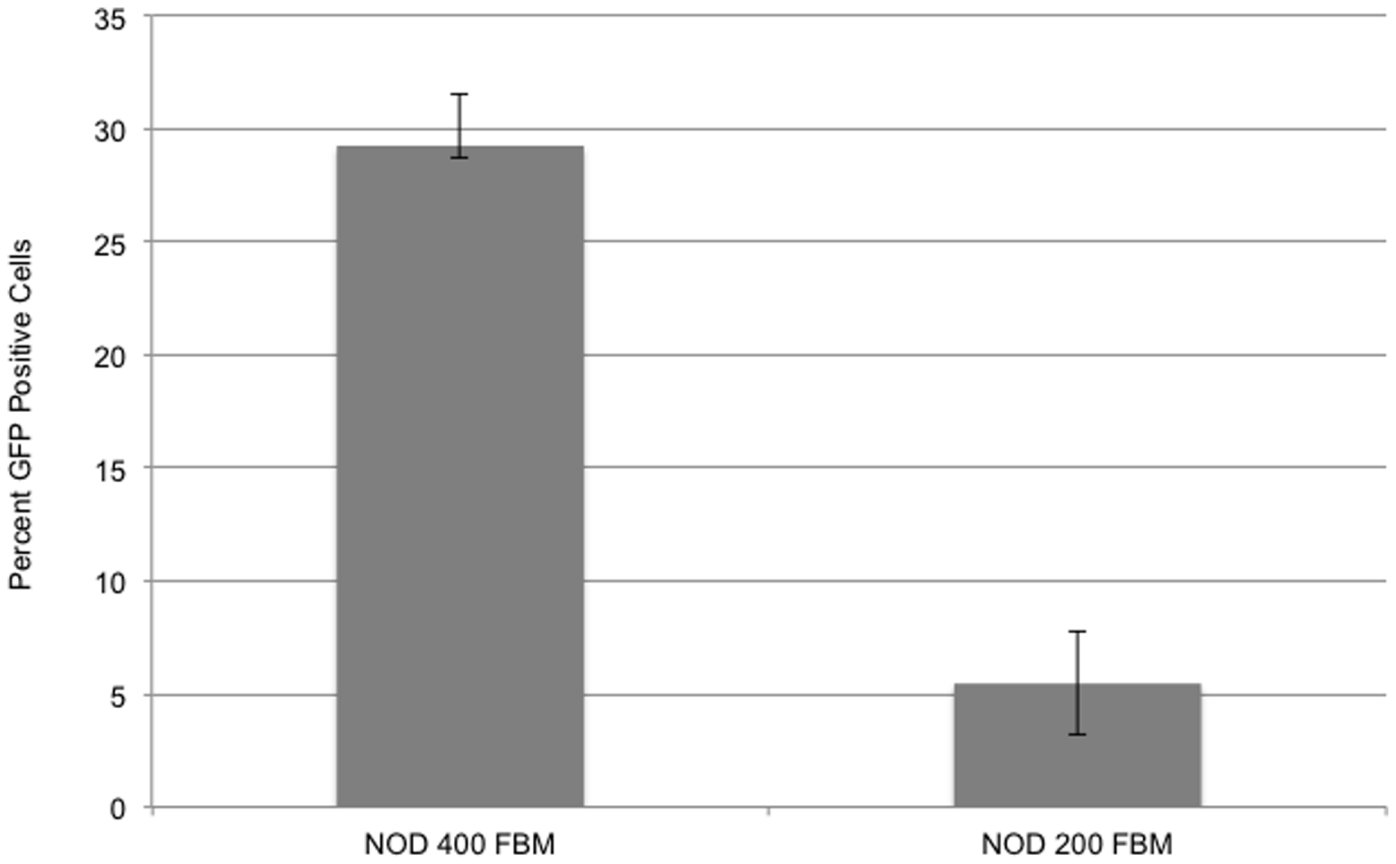
Percent chimerism as determined by the percentage of GFP positive nucleated cells within the blood. Bar graph on left represents the findings from NOD/SCID given 400 cGy gamma irradiation and treated with fractional laser and fresh bone marrow cells. Bar graph on the right represents the findings from NOD/SCID given 200 cGy gamma irradiation and treated with fractional laser and fresh bone marrow cells.

Initial experiments were carried out in using female NOD/SCID mice as recipients of GFP labeled bone marrow cells taken from donor male transgenic C57BL/6-Tg (UBC-GFP) mice. Recipient mice were irradiated with a single dose of 200 or 400 cGy gamma radiation. Laser parameters were set, (35 Millijoule [mj] with 11% density and no coagulation) to create channels of approximately 35–50 μ in depth and 7.5×10^5^ fresh total donor bone marrow cells were placed over the laser treated area. Control NOD/SCID irradiated and laser treated mice received no donor cells. Three weeks after treatment (post-irradiation) all animals that received 400 cGy irradiation but no donor cells were dead while those receiving 200 cGy irradiation but no donor cells survived. Animals receiving a lower dose of irradiation (200 cGy) and donor cells all survived (3 of 3 treated) but chimerism was low, averaging 5.46% as determined by circulating positive GFP cells (Figure 2). In animals that were treated with the higher dose of irradiation (400 cGy) and donor cells, only half (2 of 4) survived but their chimerism was significantly higher with an average of 29.25%. While these mice had significant levels of chimerism, none survived more than 3 months.

In an effort to improve on these results, we attempted to increase the depth of laser channels by increasing laser settings to 150 mJ with 22% density and no coagulation. These was done with the hope of delivering bone marrow cells deeper to better reach the circulation and have a greater chance for engraftment. We also treated some recipient NOD/SCID mice with donor cells that were depleted of lineage positive cells. Lineage negative (Lin^−^) bone marrow cells are enriched for hematopoietic stem cells. All animals were treated with single dose of 400 cGy irradiation as this had yielded the highest chimerism in previous experiments. Irradiated and laser treated mice received 1×10^6^ fresh bone marrow cells or 7.5×10^5^ Lin^−^ cells. Despite these attempts, all irradiated recipient NOD/SCID mice receiving bone marrow cells (fresh or Lin^−^) did not survive to three weeks. Positive control irradiated NOD/SCID mice receiving bone marrow cells by tail vein injection did however survive.

### Generation of EGFP Chimeric C57/BL6 Mice

We wished to further investigate the effects of radiation dose, an enriched population of hematoietic stem (Lin^−^) cells and channel depth on chimerism. This proved difficult in NOD/SCID mice due to their relative fragility, particularly in terms of radiation dosing and tissue injury. We then chose to continue these experiments with syngeneic C57/BL6 mice. We have previously reported on the successful generation of long term chimeric animals (by tail vein injection) using syngeneic C57/BL6 mice [11]. Combined radiation doses (split into two administrations) of greater than 1000 cGy were generally regarded as lethal ablation and did not yield appreciable chimerism using this approach. We therefore set out to perform partial ablation with a single dose of 400 cGy.

**Figure 3:**
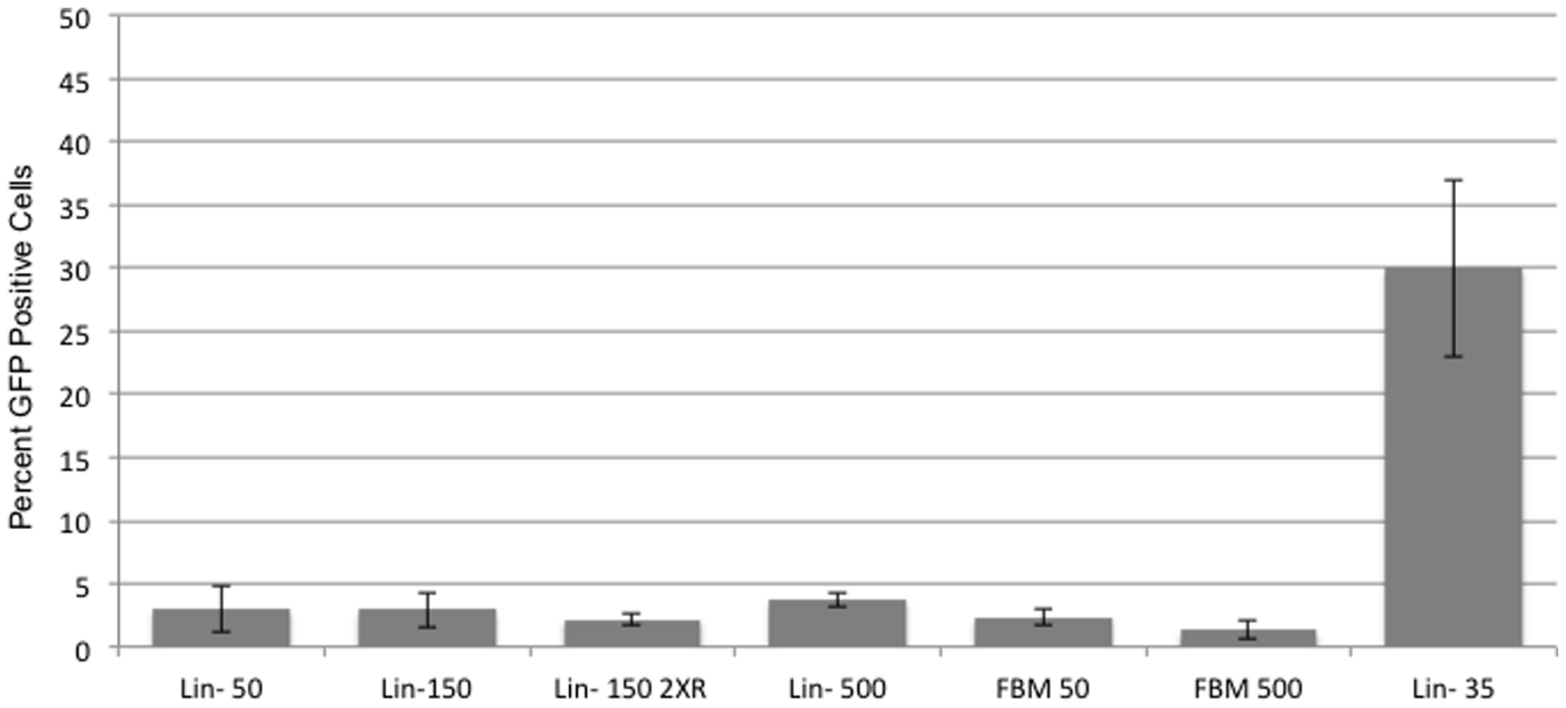
Percent chimerism as determined by the percentage of GFP positive nucleated cells within the blood of C57/BL6 mice gamma irradiated (400 cGy) after fractional laser treatment and administration of bone marrow cells. Lin- 50: Laser set at 50 mJ and Lin^−^ cells applied. Lin- 150: Laser set at 150 mJ and Lin^−^ cells applied. Lin- 150 2XR: An additional dose of 400 cGy gamma irradiation given (total of two doses), laser was set at 150 mJ, and Lin^−^ cells applied. FBM 50: Laser set at 50 mJ and fresh bone marrow cells applied. FBM 500: Laser set at 500 mJ and fresh bone marrow applied. Lin- 35: Laser set at 35 mJ and Lin^−^ cells applied. Of particular not is that these Lin- 35 treated mice remained alive and chimeric for more than one year.

We performed laser ablation to C57/BL6 mouse skin at various energy intensities with an initial goal to create channels into the skin at depths that would reach the subcutaneous vascular plexus in order for cells to reach the systemic circulation rapidly. We determined that laser conduits at depths of 350 to 500 microns (settings of 500 mJ, 22% density and no coagulation) would reach this plexus. Animals receiving this dosage however exhibited very little chimerism regardless of whether they were administered fresh bone marrow or lineage negative cells (Figure 3). In reducing the energy settings of the laser to the 50 or 150 mJ settings, there was only marginal improvement in chimerism. Adding an additional dose of (400 cGy) radiation also failed to produce a benefit in higher chimerism.

**Figure 4:**
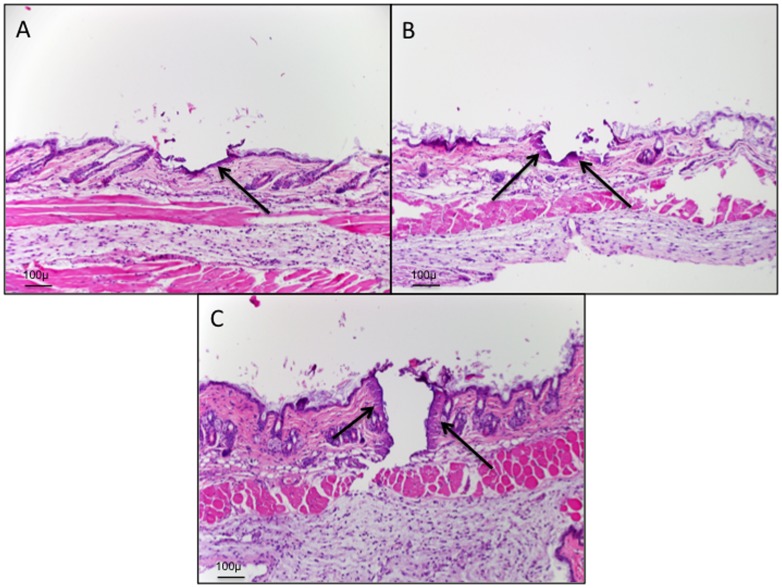
Histologic sections following laser treatment. A) C57/BL6 mouse skin treated with fractional Erb:YAG laser at the 35 μ setting (A), 50 μ setting (B) and 150 μ setting (C). There is increasing amounts of homogenization of the collagen (indicated by the arrows) at the edge of channels with greater applied laser energy and increased channel depth. The areas of collagen homogenization represent thermal damage.

When we utilized the 35 mJ setting (as in our first experiments with NOD/SCID mice) we were able to achieve appreciable chimerism in mice given a single dose of 400 cGy radiation followed by the administration of lineage negative donor cells. Unlike the immune compromised mice however, these mice remained alive for more than a year and continued to remain chimeric (Figure 3). It was however perplexing that similar mice treated with the 50 mJ setting did not experience higher chimerism. In examining the histology of C57/BL6 mouse skin treated with these settings it did not seem that channel depth was appreciably different in terms of reaching specific tissue landmarks. The primary difference appeared to be in the amount of thermal damage or coagulation at the borders of the laser conduits. It is then possible that administered cells could have been inhibited to reach systemic circulation by a region of denatured collagen (Figure 4).

We next examined the possibility that there may be a greater benefit in attempting to deliver bone marrow cells through larger channels. Chambers containing lineage negative or fresh bone marrow cells were placed over 4 mm fresh full thickness wounds (made using a standard 4 mm punch biopsy tool). All mice had been previously irradiated with 400 cGy as was performed in laser treated animals. No animals survived at 21 days.

### Analysis of Cell Distribution

In mice exhibiting low chimerism we attempted to determine if the low numbers of cells observed by FACS analysis was in part due to the loss of GFP expression in circulating donor cells. Quantitative PCR analysis of GFP and Sry DNA extracted from circulating cells demonstrated that this was not likely to be the case. Controls were peripheral blood from GFP and C57BL/6 mice.

**Figure 5:**
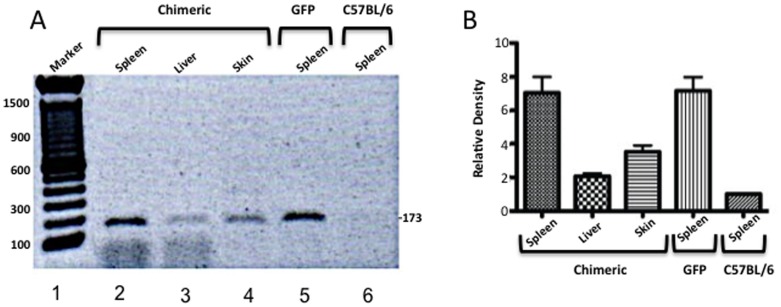
PCR of tissues derived from chimeric and control mice. PCR performed with GFP specific forward (5′-AAG TTC ATC TGC ACC ACC G- 3′) and reverse (5′-TCC TTG AAG AAG ATG GTG CG-3′) primers. (A) Representative gel of GFP PCR products. Lane 1: MW Marker. Lanes 2, 3, & 4: PCR products derived from spleen the (Lane 2), liver (Lane 3) and skin (Lane 4) of a mouse administered Lin^−^ bone marrow cells following fractional laser treatment. This animal exhibited 21.1% blood chimerism. Lane 5: PCR product from GFP transgenic mouse. Lane 6: Absence of PCR product in control C57/BL6 mouse. (B) Bar graph representation of relative expression of several (3 per group) GFP in chimeric (treated with fractional laser and Lin^−^ bone marrow cells), GFP (transgenic) and control C57/BL6 mice. Chimeric mice exhibited 5–21.1% blood chimerism.

**Figure 6:**
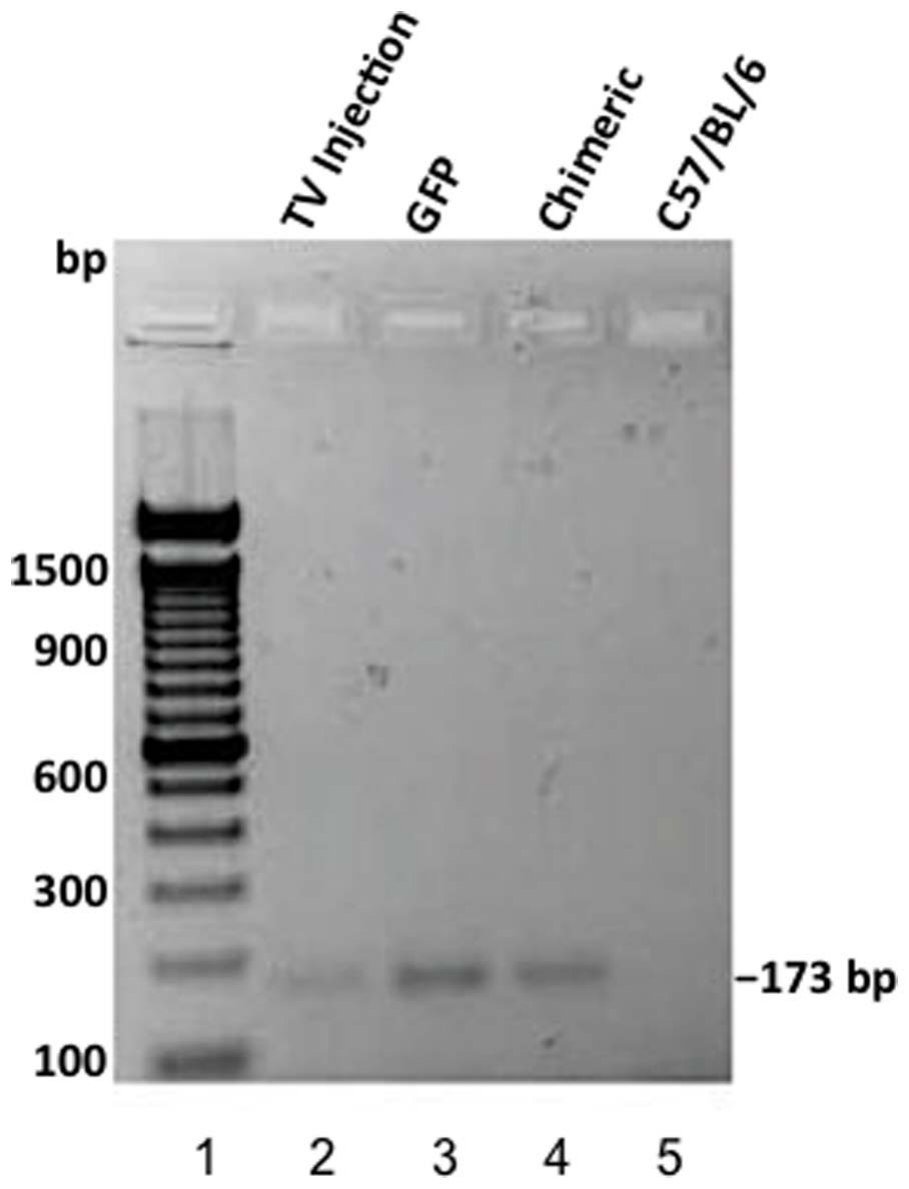
PCR for presence of GFP sequence in bone marrow cells in culture derived from chimeric and control mice. Lane 1: MW Marker. Lanes 3: Control irradiated mouse given Lin^−^ bone marrow cells by tail vein injection. This mouse had 70% blood chimerism. Lane 3: Transgenic (donor) GFP mouse. Lane 4: Chimeric mouse treated with fractional laser and Lin^−^ bone marrow cells. This mouse had 39.9% chimerism and survived more than one year. Lane 5: Control C57/BL6 mouse.

To better investigate the fate of the transplanted GFP cells, necropsies were performed on the chimeric mice 2 months after transplantation. Skin spleen and liver were examined for the presence of GFP cells by PCR. All tissue had varying amounts of GFP cells (Figure 5) with the highest signal being found in spleen. This could indicate that donor cells may first traffic to and engraft in the spleen. It also was important for us to determine if cells detected in the marrow had truly engrafted or were just trafficking through. We then placed bone marrow from treated animals in culture for 2 weeks to eliminate any transiently trafficking or differentiated cells. PCR analysis of these cultures clearly indicated the presence of engrafted donor cells (Figure 6).

## Discussion

In this report we have utilized a bone marrow transplantation model to demonstrate that fractional lasers can be used to assist in the percutaneous delivery of stem cells. The delivered cells were capable of reaching distant sites, remain functional and reconstitute organ function. While it is possible that fractional laser mostly provides only a breach in the barrier function of the skin, there are other points to consider. Efforts to deliver stem cells and create chimeric animals failed when lin^−^ cells were applied to larger, surgically excised areas created with a standard punch biopsy tool. This failure to achieve chimerism occurred despite the fact that punch biopsy wounds provided a much deeper channel which could hold a substantially greater volume of delivered cells in suspension than the (cumulative) channels created by fractional laser. This provided evidence that ablative fractional lasers might have other advantages for cell delivery beyond the ability to breach the barrier function of skin.

Given some of our observations, it is possible that laser created channels have an affinity to recruit particular cells. While the percentage of stem cells capable of reconstituting hematopoiesis is enhanced following lineage depletion of fresh bone marrow, their numbers are still quite rare [12]. Also, the amount of cells that could enter a laser created channel (measuring approximately 100 μ in diameter and 35 μ in depth) by simple diffusion would be expected to be at best restricted. Yet despite these limitations, we were able to create stable chimeras. Conduits created by ablative fractional lasers may then likely have distinct biologic features that can overcome these obstacles.

Treatment with ablative fractional lasers alone can induce a biologic response as has been widely demonstrated in reports where these have been used to reduce scars, photodamage and rhytides [13]–[15]. The conduits produced by fractional lasers are created by a unique, selective vaporization of tissue without the need for debridement; nor is there significant associated thermal damage. This type of minute tissue ablation thus differs significantly from injuries commonly occurring in nature. Studies of ablative fractional lasers have reported stimulation of tissue remodeling and the release of cytokines that appear to be distinct from other forms of treatment or injury [16], [17]. Some of these released cytokines, such as Wnt5a, are active in several pathways involving cell adhesion and migration [16]. These factors are could have played important in recruiting stem cells to generate chimerics in our experiments.

Another surprising finding was that the increasing the depth of laser channels seemed to correlate with a reduced ability to create chimeric animals. This could have been due to the increased amount of thermal damage observed with increasing energies to create deeper channels. The larger area of thermal damage could have created a new barrier that would prevent the ability of cells within the laser channels to reach systemic distribution. Other possibilities include an altered cytokine response associated with the creation of deeper channels and/or administering higher energies to the skin. Further studies beyond the scope of this report will be required to answer these questions and determine the optimal energy parameters for fractional laser assisted delivery of cells and other materials.

The findings presented in this report do however support the supposition that a percutaneous means of cell and drug delivery can be accomplished through small ablative fractional laser channels where the delivered materials do not appear to be limited to materials having restricted size and solubility profiles. In our experiments this could not be accomplished by the direct application of cells after the surgical removal of skin. While the authors are not suggesting that delivery of hematopoietic stem cells via ablative fractional laser be used as a substitute for current bone marrow transplantation techniques, our findings do provide proof of principle that functional stem cell delivery at distant sites can be accomplished by these means. Ablative fractional lasers are however likely to application for the non-invasive delivery of functional cells to treat other local and systemic processes.
